# Combined flow-based imaging assessment of optimal cardiac resynchronization therapy pacing vector: a case report

**DOI:** 10.1186/s13256-019-2048-1

**Published:** 2019-05-25

**Authors:** A. R. Martiniello, V. Bianchi, G. Tonti, C. Cioppa, V. Tavoletta, A. D’Onofrio, V. M. Caso, G. Pedrizzetti, P. Caso

**Affiliations:** 1 Cardiology Division, Monaldi Hospital, AORN Ospedali dei Colli, Via Leonardo Bianchi, 80131 Napoli, Italy; 2 Cardiology Division, University of Chieti, Chieti, Italy; 3 Department of Engineering and Architecture, University of Trieste, Trieste, Italy

**Keywords:** Mitral regurgitation, Three-dimensional echocardiography, Full-volume color Doppler echocardiography, Echo-PIV, Cardiac resynchronization therapy

## Abstract

**Background:**

There are still many pendent issues about the effective evaluation of cardiac resynchronization therapy impact on functional mitral regurgitation. In order to reduce the intrinsic difficulties of quantification of functional mitral regurgitation itself, an automatic quantification of real-time three-dimensional full-volume color Doppler transthoracic echocardiography was proposed as a new, rapid, and accurate method for the assessment of functional mitral regurgitation severity. Recent studies suggested that images of left ventricle flow by echo-particle imaging velocimetry could be a useful marker of synchrony. Echo-particle imaging velocimetry has shown that regional anomalies of synchrony/synergy of the left ventricle are related to the alteration, reduction, or suppression of the physiological intracavitary pressure gradients.

**Case summary:**

We describe a case in which the two technologies are used in combination during acute echocardiographic optimization of left pacing vector in a 63-year-old man, Caucasian, who showed worsening heart failure symptoms a few days after an implant, and the effect of the device’s optimization at 6-month follow-up.

**Discussion:**

The degree of realignment of hemodynamic forces, with quantitative analysis of the orientation of blood flow momentum (*φ*), can represent improvement of fluid dynamics synchrony of the left ventricle, and explain, with a new deterministic parameter, the effects of cardiac resynchronization therapy on functional mitral regurgitation. Real-time three-dimensional color flow Doppler quantification is feasible and accurate for measurement of mitral inflow, left ventricular outflow stroke volumes, and functional mitral regurgitation severity.

**Conclusion:**

This clinical case offers an innovative and accurate approach for acute echocardiographic optimization of left pacing vector. It shows clinical utility of combined three-dimensional full-volume color Doppler transthoracic echocardiography/echo-particle imaging velocimetry assessment to increase response to cardiac resynchronization therapy, in terms of reduction of functional mitral regurgitation, improving fluid dynamics synchrony of the left ventricle.

**Electronic supplementary material:**

The online version of this article (https://doi.org/10.1186/s13256-019-2048-1) contains supplementary material, which is available to authorized users.

## Introduction

The role of echocardiographic imaging is essential in the follow-up of patients after cardiac resynchronization therapy (CRT). Dysfunction of the device can be discovered by echo control with a multiparametric approach. This is the first case report in which automatic quantification of real-time three-dimensional full-volume color Doppler transthoracic echocardiography (FVCD) has been proposed as a new, rapid, and accurate method for the assessment of functional mitral regurgitation (FMR) severity pre-CRT and post-CRT, in combination with fluid dynamic approach using echo-particle imaging velocimetry (echo-PIV) technique.

Reduction in FMR is one of the mechanisms by which CRT exerts its beneficial effects. This innovative and combined approach can allow a more effective optimization of the biventricular device at follow-up. The quantification of mitral insufficiency during acute study can be associated with the quantitative analysis of the orientation of blood flow momentum (*φ*), which represents a new deterministic parameter of fluid dynamics synchrony. The combined approach can explain the effects of CRT on the realignment of hemodynamic forces involved in the pathophysiologic determinants of FMR. The short time of post-processing during acute study would increase the cultural interaction between electrophysiologist and echocardiographer, with lower costs required than those necessary for invasive monitoring of cardiac performance that can be used only during implantation of the device.

## Case presentation

**Fig. 1:**
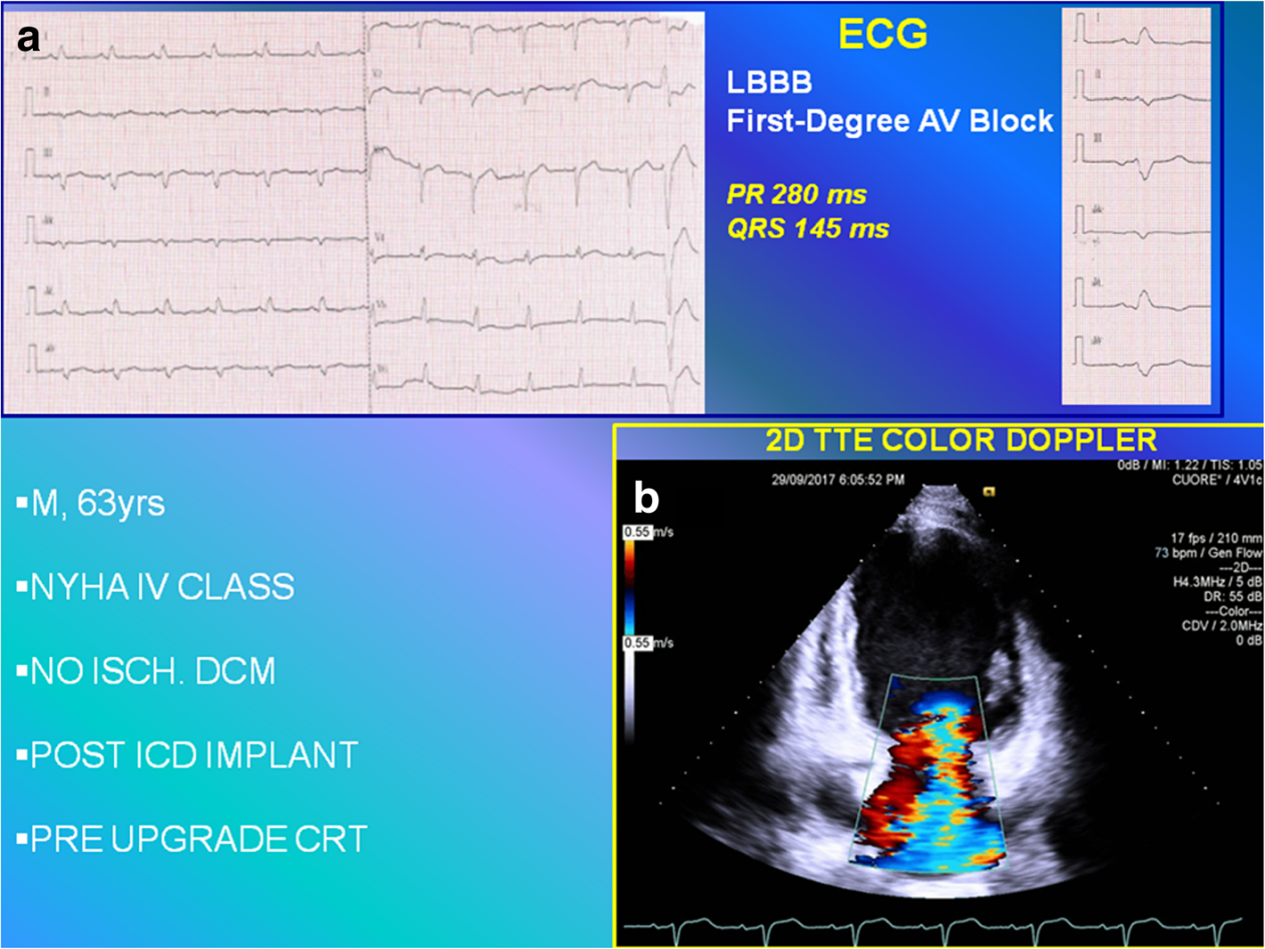
**a** Twelve-lead electrocardiogram showing the wide native QRS complex (145 ms) with first-degree atrioventricular block (PR 280 ms). **b** Two-dimensional color transthoracic echocardiogram apical four-chamber view. *AV* atrioventricular, *CRT* cardiac resynchronization therapy, *ECG* electrocardiogram, *ICD* implantable cardioverter defibrillator, *LBBB* left bundle branch block, *M* male, *NO ISCH. DCM* non-ischemic dilated cardiomyopathy, *NYHA* New York Heart Association, *TTE* transthoracic echocardiogram


We describe the case of a 63-year-old man, Caucasian, affected by non-ischemic dilated cardiomyopathy who did not drink alcohol, did not smoke tobacco, and did not have diabetes. He had an implantable cardioverter defibrillator implanted, in New York Heart Association (NYHA) IV class, and left bundle branch block (LBBB; QRS duration of 145 ms). He was referred for CRT-D upgrade, awaiting cardiac transplantation, despite optimal medical therapy: b-Blockade, loop-diuretic, angiotensin-converting enzyme (ACE) inhibitor, K-sparing agent, and ivabradine. Standard clinical imaging protocol revealed a dilated left ventricle with an end-systolic volume (ESV) of 380 ml, an ejection fraction (EF) of 4.8% as measured by the modified Simpson’s method, and severe FMR, assessed by qualitative estimation with two-dimensional color flow Doppler approach, showing a very large central jet and reaching the posterior wall of the left atrium (see Fig. 1 and Additional file 1: Video S1).

**Additional file 1:** **Video S1.** Transthoracic echocardiogram apical four-chamber view showing a dilated left ventricular pre-implant, with severe functional mitral regurgitation, assessed by qualitative estimation with two-dimensional color flow Doppler approach. (WMV 1610 kb)


He underwent the implant of a CRT-D device with a quadripolar left ventricular (LV) lead placed in the posterolateral branch of the coronary sinus. After recording the right ventricle (RV)-to-LV electrical delay at each of the four LV rings, we chose the A1 unipolar vector for LV pacing (greatest electrical delay 80 ms).

**Fig. 2:**
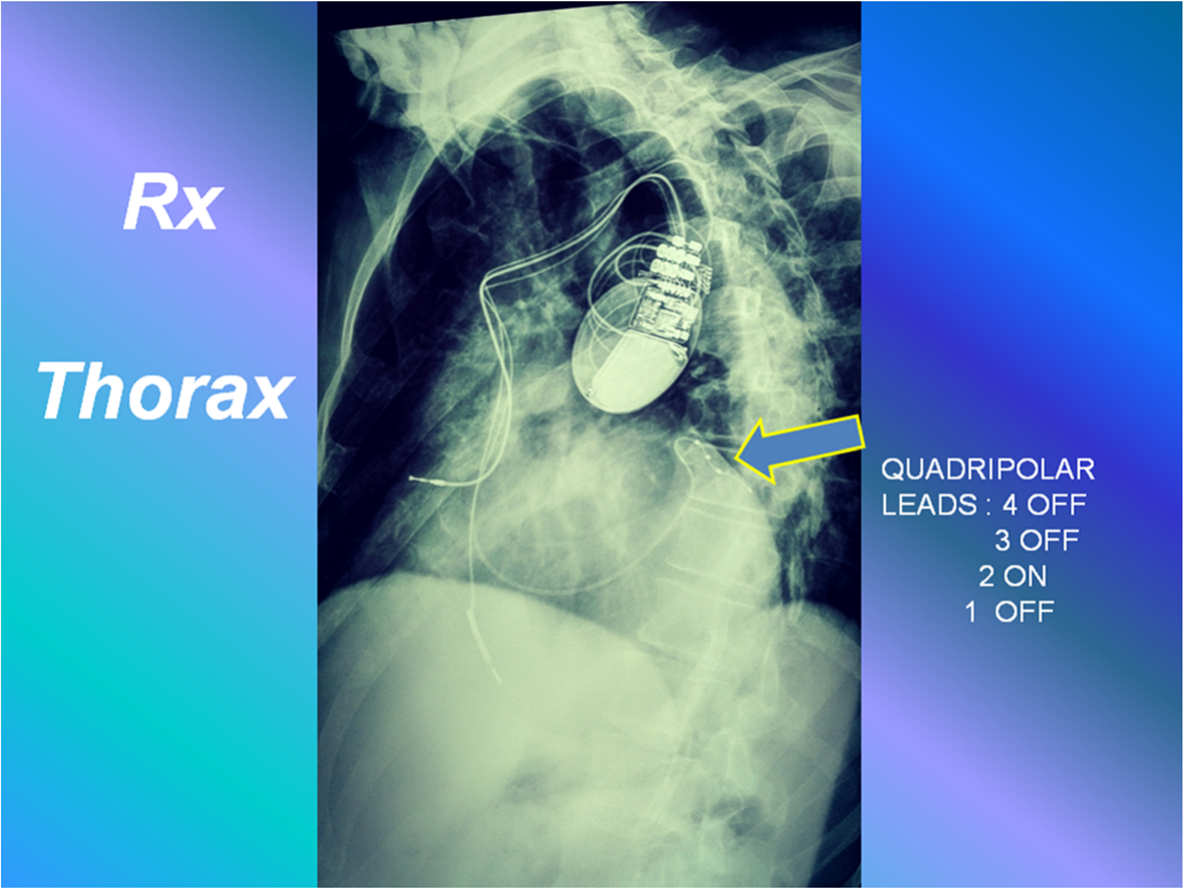
Left anterior oblique chest X-ray view displaying the left ventricular quadripolar lead (*arrow*)

At 13-day post-implant follow-up, he showed worsening heart failure (HF) symptoms and only A2 unipolar LV vector configuration, with interventricular (VV) interval of 0 ms, was suitable for simultaneous biventricular activation (Fig. 2).

Echo-PIV was then used, during the acute study with contrast agent bubbles, to evaluate the orientation and relative magnitude of blood-induced intraventricular forces in correspondence of different pacing settings.

**Fig. 3:**
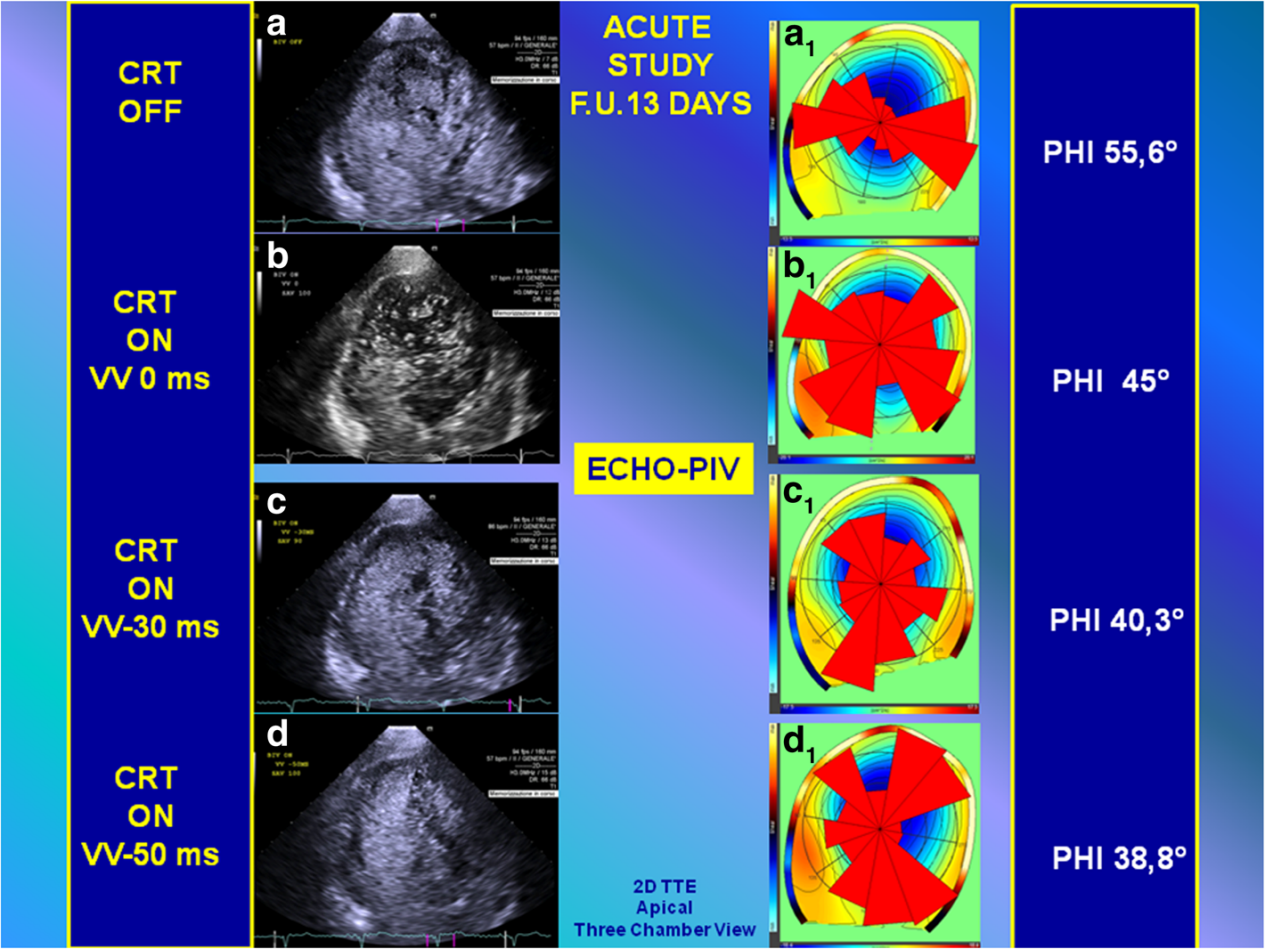
Quantitative analysis (to compare with the results of analysis represented in Fig. 7) by echo-particle imaging velocimetry of the orientation angle (*φ*) of the global hemodynamic forces exchanged between blood and surrounding tissues during acute study (post-cardiac resynchronization therapy 13-day follow-up). *Left*: Two-dimensional transthoracic echocardiogram high-temporal resolution contrast echocardiographies are performed by three-chamber apical view approach during acute study of different setting (**a**, **b**, **c**, **d**). *Right*: Changes in electrical activation settings modify the orientation of intraventricular forces during acute study. The intraventricular forces were predominantly transverse and not aligned along the LVaxis (**a**_**1**_) as quantified by the large value of their mean angle φ (φ = 55.6°). A first setting option (CRT ON, VV delay 0 ms) changed the orientation of intraventricular forces (**b**_**1**_) reducing the angle (φ = 45°), and increasing the delay (CRT ON, VV delay − 30 ms) improved the alignment (**c**_**1**_) reducing the angle (φ = 40.3°). The sequential biventricular activation with delay − 50 ms (**d**_**1**_) provided the best alignment of intraventricular forces (φ = 38.8°). *CRT* cardiac resynchronization therapy, *F.U.* follow-up, *PIV* particle imaging velocimetry, *VV* interventricular, *TTE* transthoracic echocardiogram


Without pacing stimulation (CRT OFF, Fig. 3a, and Additional file 2: Video S2) the intraventricular flow was dominated by rotation without evident inflow–outflow dynamics. As a result the intraventricular forces were predominantly transverse and not aligned along the LV axis (Fig. 3a_1_) as quantified by the large value of their mean angle *φ* (*φ* = 55.6°, this angle ranges from 0°, when forces are aligned with the LV axis, to 90°). A first setting option (CRT ON, VV delay 0 ms, Fig. 3b and Additional file 3: Video S3) changed the orientation of intraventricular forces (Fig. 3b_1_) reducing the angle (*φ* = 45°), and increasing the delay (CRT ON, VV delay − 30 ms, Fig. 3c and Additional file 4: Video S4) improved the alignment (Fig. 3c_1_) reducing the angle (*φ* = 40.3°). Eventually, the sequential biventricular activation with delay − 50 ms (Fig. 3d and Additional file 5: Video S5) provided the best alignment of intraventricular forces (Fig. 3d_1_, *φ* = 38.8°).

**Additional file 2:** **Videos S2.** Two-dimensional contrast-enhanced cine loops with a particle image velocimetry technique for different pacing settings: cardiac resynchronization therapy-OFF (Video 2). (WMV 692 kb)



**Additional file 3:** **Video S3.** Cardiac resynchronization therapy ON with interventricular delay 0 ms (Video 3). (WMV 4820 kb)



**Additional file 4:** **Videos S4.** Cardiac resynchronization therapy ON with interventricular delay − 30 ms (Video 4). (WMV 247 kb)



**Additional file 5:** **Video S5.** Cardiac resynchronization therapy ON with interventricular delay − 50 ms (Video 5). (WMV 645 kb)


**Fig. 4:**
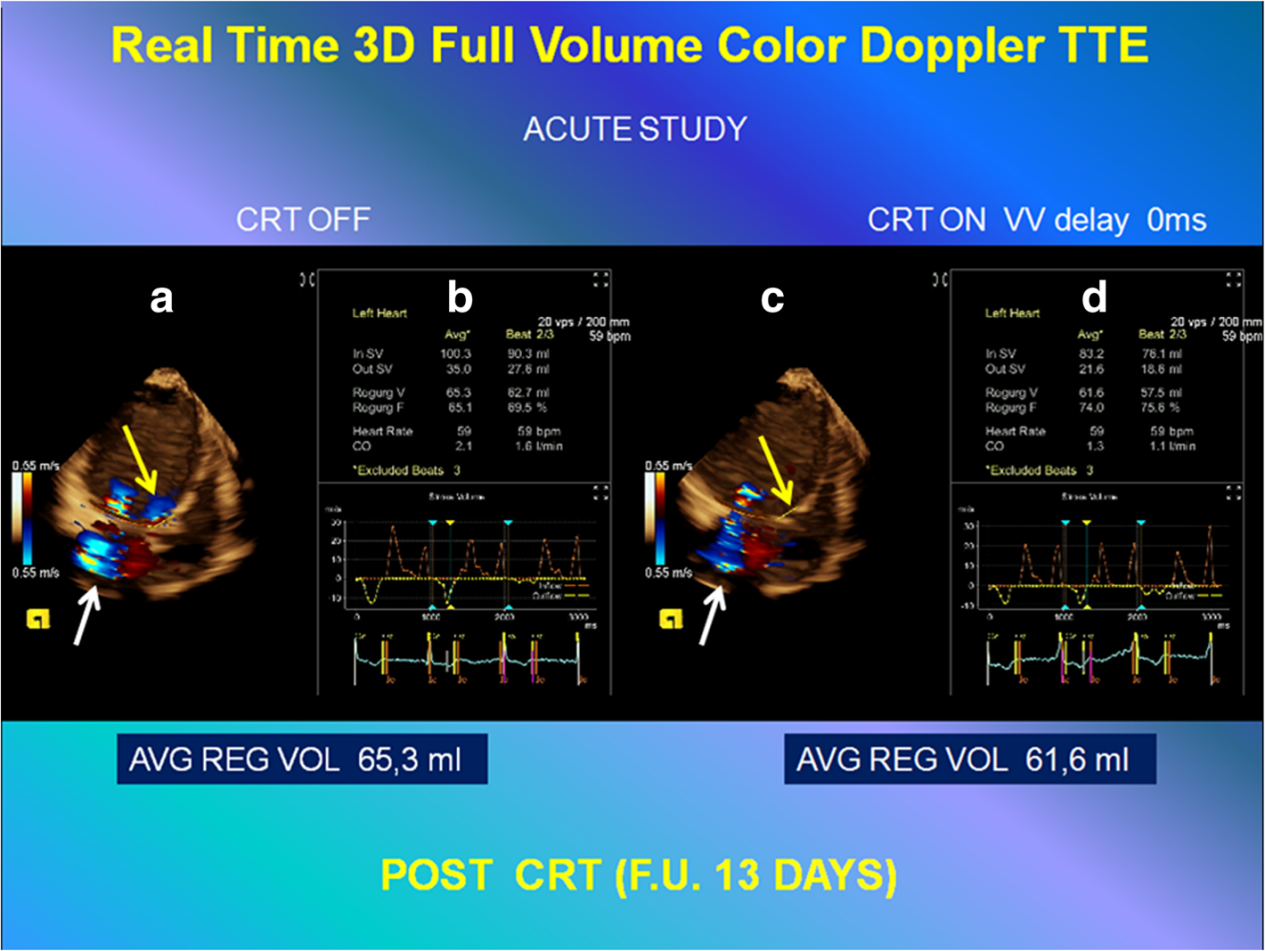
Quantitative analysis (to compare with the results of analysis represented in Fig. 6), of functional mitral regurgitation by three-dimensional full-volume color Doppler transthoracic echocardiography: acute study (post-cardiac resynchronization therapy 13-day follow-up). *Left*: Setting cardiac resynchronization therapy OFF. *Right*: Setting cardiac resynchronization therapy ON with interventricular delay 0 ms. **a**, **c** Automated anatomy detection of the left ventricular endocardial border, mitral annulus, left ventricular outflow, and placement of three-dimensional hemispheric flow sampling planes in the mitral annulus (*white arrow*) and left ventricular outflow (*yellow arrow*). **b**, **d** Flow-time curve derived from automatic flow volume aggregation at each frame in the cardiac cycle and the automated calculation of the mitral (*In SV*), aortic (*Out SV*) stroke volume, regurgitant volume, regurgitation fraction (*RF*), and cardiac output (*CO*), with mean value, in three cardiac cycles, respectively. *AVG* mean value, *CRT* cardiac resynchronization therapy, *F.U.* follow-up, *REG VOL* regurgitant volume, *TTE* transthoracic echocardiogram, *VV* interventricular


No reduction of FMR by three-dimensional FVCD, during the same acute study with shutdown versus reactivation of device, was demonstrated, as shown in Figure 4 and by comparing Additional file 6: Video S6 and Additional file 7: Video S7.

**Additional file 6:** **Videos S6.** Real-time three-dimensional color flow Doppler quantification at 13-day follow-up, during acute study: cardiac resynchronization therapy OFF (Video 6). (WMV 559 kb)



**Additional file 7:** **Video S7.** Cardiac resynchronization therapy ON with interventricular delay 0 ms (Video 7). (WMV 559 kb)


The data acquisition time, by three-chamber apical view, for each three-dimensional color Doppler data set was approximately 5 seconds, and it took less than 3 minutes to analyze the average regurgitation volume, with automated anatomy detection of the LV endocardial border, mitral annulus (MA), LV outflow (LVOT), and placement of three-dimensional hemispheric flow sampling planes in the MA and LVOT. The software of three-dimensional FVCD computed the flow volumes as the area under the curve of both the MA and LVOT flow in three cardiac cycles, and FMR volume was calculated by subtracting LVOT stroke volume from MA stroke volume.

### Results at 6-month follow-up

**Fig. 5:**
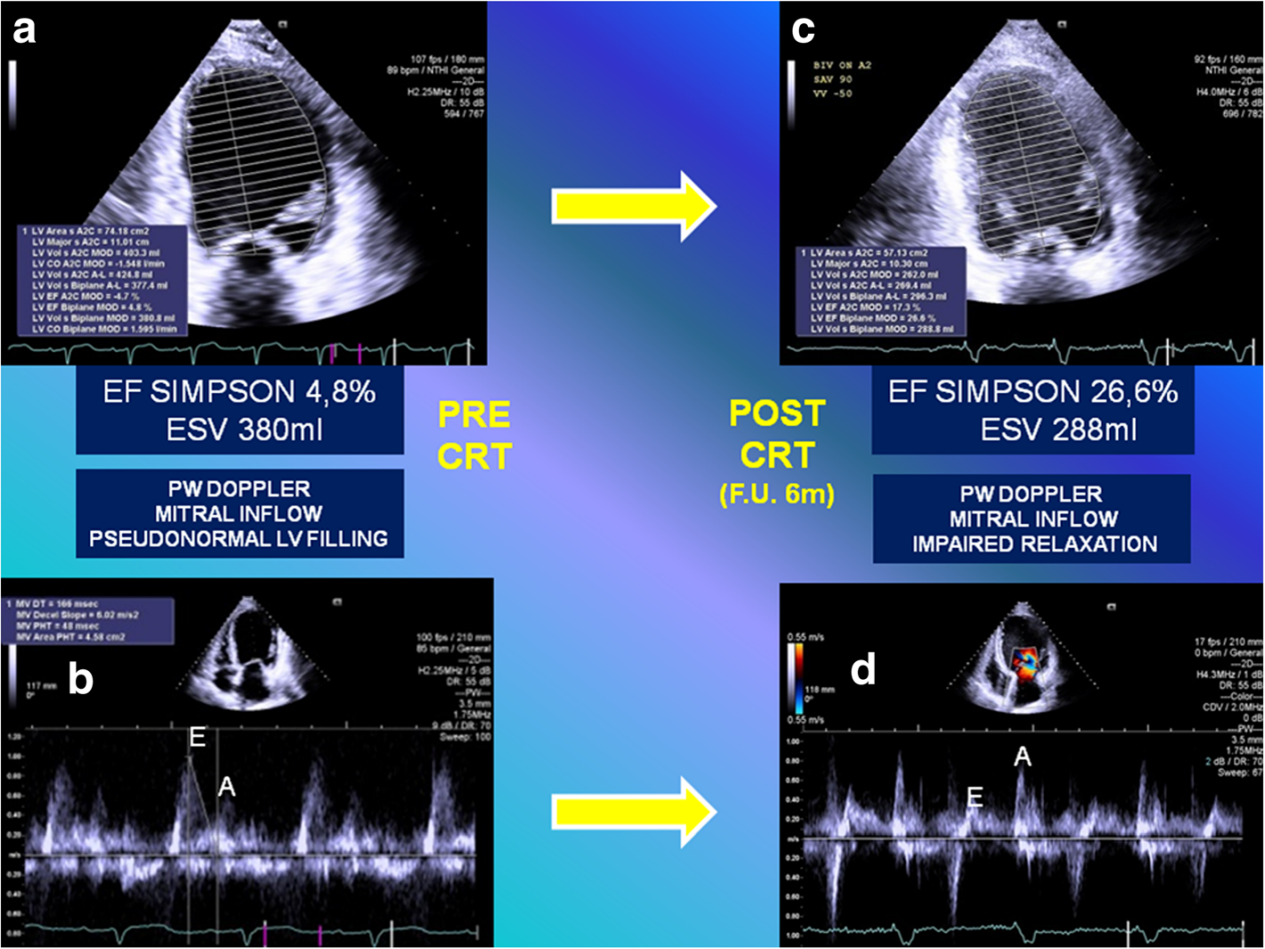
*Left side*: **a** Dilated left ventricle with an end-systolic volume of 380 ml, and an ejection fraction of 4.8% as was measured by the modified Simpson’s method pre-implant. **b** Pulsed wave Doppler mitral inflow pattern showing advanced diastolic dysfunction (deceleration time (*DT*) of early filling velocity, 165 ms). *Right side*: **c** Dilated left ventricle with reduced end-systolic volume of 288 ml and improved ejection fraction of 26.6% as was measured by the modified Simpson’s method post-implant at 6-month follow-up. **d** Pulsed wave Doppler mitral inflow pattern showing impaired relaxation. *CRT* cardiac resynchronization therapy, *EF* ejection fraction, *ESV* end-systolic volume, *F.U.* follow-up, *LV* left ventricle, *PW* pulsed wave

**Fig. 6:**
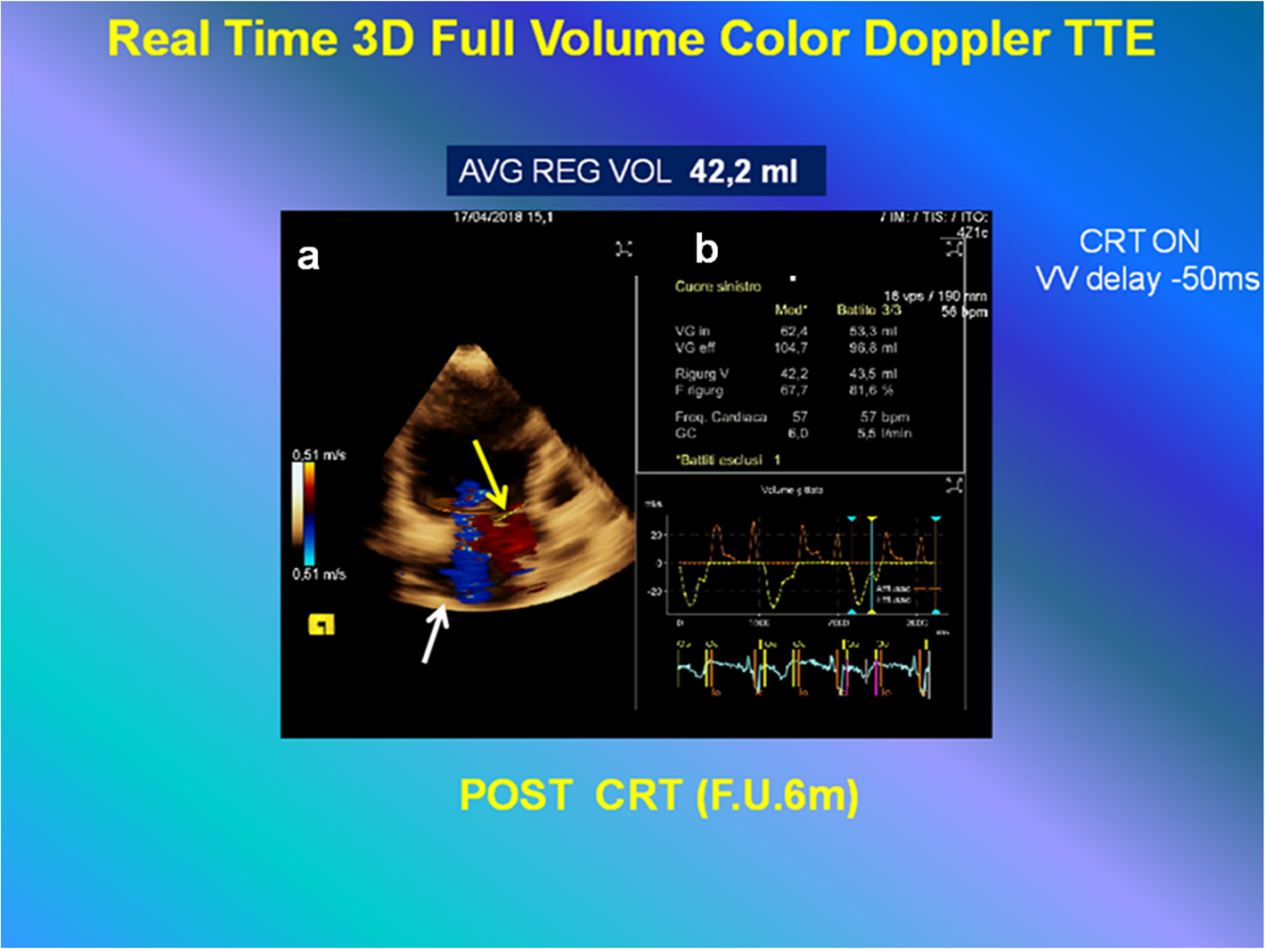
Quantitative analysis (to compare with the results of analysis represented in Fig. 4), of functional mitral regurgitation by three-dimensional full-volume color Doppler transthoracic echocardiography: at 6-month follow-up setting cardiac resynchronization therapy ON interventricular delay − 50 ms. **a** Automated anatomy detection of the left ventricular endocardial border, mitral annulus, left ventricular outflow, and placement of three-dimensional hemispheric flow sampling planes in the mitral annulus (*white arrow*) and left ventricular outflow (*yellow arrow*). **b** Flow-time curve derived from automatic flow volume aggregation at each frame in the cardiac cycle and the automated calculation of the mitral (*In SV*), aortic (*Out SV*) stroke volume, regurgitant volume, regurgitation fraction (*RF*), and cardiac output (*CO*), with mean value, in three cardiac cycles, respectively. *AVG* mean value, *CRT* cardiac resynchronization therapy, *F.U.* follow-up, *REG VOL* regurgitant volume, *VV* interventricular

Our patient showed an improvement of NYHA class (III versus IV) and LV EF (26.6% versus 4.8%). Significant reduction of ESV (288 ml versus 380 ml) and persistent improvement of diastolic function were obtained. The regularized function is noticeable in Additional file 8: Video S8 (to be compared with Additional file 1: Video S1) and it is summarized in Fig. 5. At follow-up, a significant reduction of FMR (mean value regurgitant volume, 42.2 ml versus 65.3 ml) was estimated (Fig. 6, Additional file 9: Video S9, Table 1).

**Table 1:** Profile three-dimensional full-volume Color Doppler of echocardiographic results. The last three rows are the de-aliased mitral inflow, left ventricular outflow, and mitral regurgitation volumes based on the sampled volumetric color Doppler data by three-dimensional full-volume color Doppler transthoracic echocardiography

	Acute study (13-day follow-up)	Acute study (13-day follow-up)	Acute study (6-month follow-up)
Mean value measure (ml)	CRT OFF	CRT ON VV delay 0 ms	CRT ON VV delay − 50 ms
Three-dimensional full-volume color Doppler transthoracic echocardiography: mitral inflow volume	100.3	83.2	62.4
Three-dimensional full-volume color Doppler transthoracic echocardiography left ventricular outflow: outflow volume	35	21.6	104.7
Three-dimensional full-volume color Doppler transthoracic echocardiography: mitral regurgitation volume	65.3	61.6	42.2

*CRT* cardiac resynchronization therapy, *VV* interventricular


**Additional file 8:** **Video S8.** Transthoracic echocardiogram apical four-chamber view, at 6-month follow-up showing a dilated left ventricle post-implant, with moderate-to-severe functional mitral regurgitation, assessed by qualitative estimation with two-dimensional color flow Doppler approach, with a smaller central jet and no reaching of the posterior wall of the left atrium. (WMV 231 kb)



**Additional file 9:** **Video S9.** Real-time three-dimensional color flow Doppler quantification at 6-month follow-up. (WMV 638 kb)


**Fig. 7:**
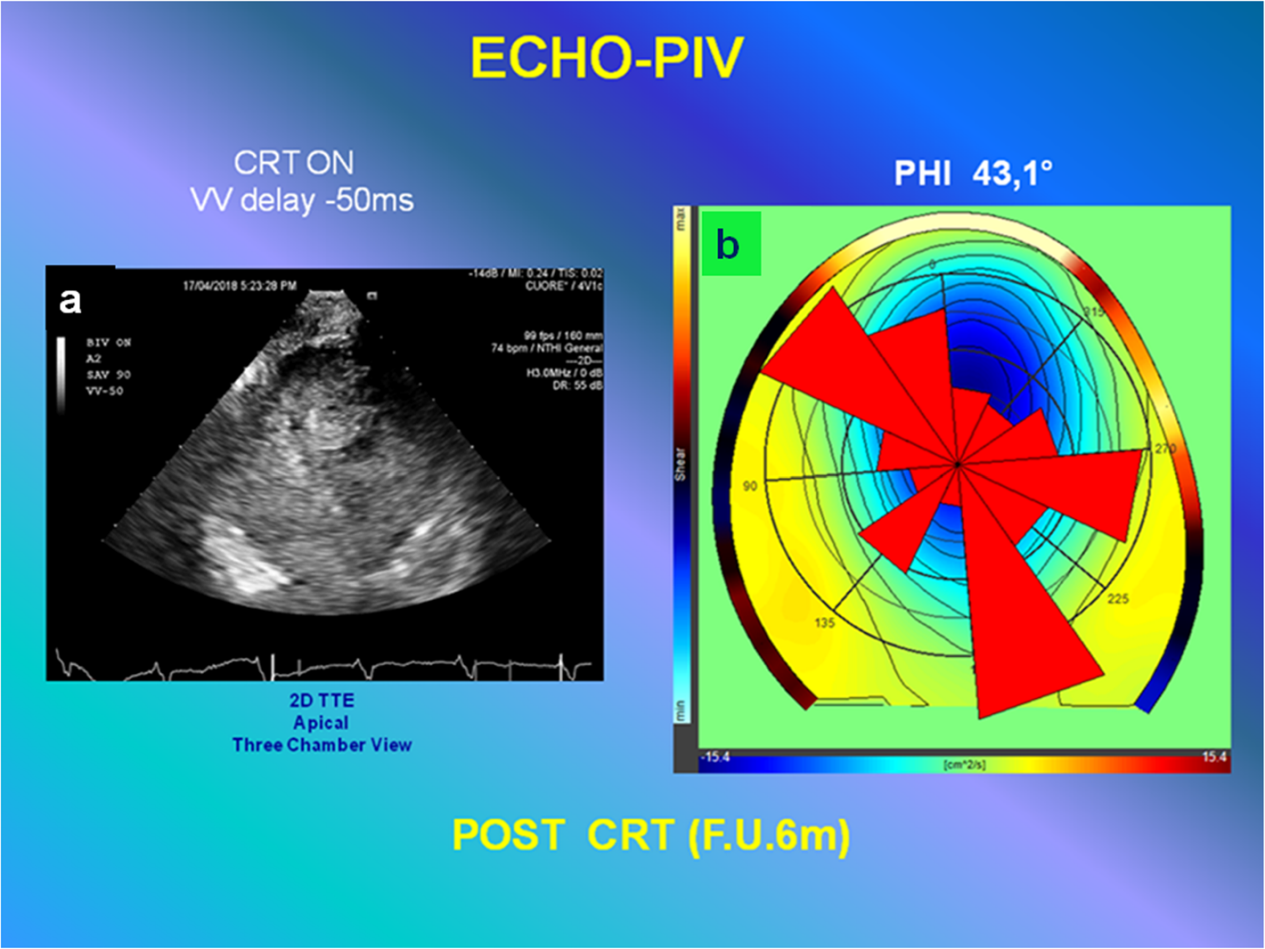
Quantitative analysis (to compare with the results of analysis represented in Fig. 3), by echo-particle imaging velocimetry of the orientation angle (*φ*) of the global hemodynamic forces exchanged between blood and surrounding tissues during acute study (post-cardiac resynchronization therapy 6-month follow-up). **a** Two-dimensional transthoracic echocardiogram high-temporal resolution contrast echocardiography is performed by three-chamber apical view approach (see Additional file 10: Video S10 interventricular − 50 ms SONOVUE 6-month follow-up). **b** The polar histogram showed with setting interventricular delay − 50 ms the most aligned intraventricular forces that were partially dominated by the longitudinal path of pressure gradient (lower *φ*, value 43.1°). *CRT* cardiac resynchronization therapy, *F.U.* follow-up, *PIV* particle imaging velocimetry, *TTE* transthoracic echocardiogram, *VV* interventricular


The intraventricular forces estimated by echo-PIV were still partially dominated by the longitudinal path of pressure gradient (Fig. 7 and Additional file 10: Video S10) with *φ* = 43.1°.

**Additional file 10:** **Video S10.** Two-dimensional contrast-enhanced cine loops with a particle image velocimetry technique. (WMV 2920 kb)


## Discussion

The availability of pacing configurations offered by quadripolar LV leads could improve patients’ response to CRT, in terms of reduction of FMR, by improving cardiac synchrony; however, the selection of an optimal setting remains a challenge, and the correct quantitative evaluation of FMR suffers from well-known pitfalls and systematic inaccuracy. The need for shape assumptions and the inability to account for the dynamic regurgitant orifices are technical limitations to two-dimensional proximal isovelocity surface area (PISA)-based effective regurgitant orifice area (EROA) and regurgitant volume measurements, especially in patients with FMR where the regurgitant orifice is thought to be largest at the beginning and end of systole, and smallest in the middle. Pulsed wave (PW) Doppler-based flow quantification techniques with two-dimensional transthoracic echocardiogram have been used to measure mitral inflow and aortic stroke volumes. This information is also used in computing flow-derived valve area in stenotic valve disease and regurgitant volume and fraction in valvular regurgitation. We know that the assumption of circular geometry of LVOT is erroneous and affects the calculation of aortic valve area by continuity equation, and the effect of spatially non-uniform flow on the continuity equation has not been clearly evaluated. Small errors in measurements of LVOT or mitral annular diameter are squared in the computation of stroke volume, and may lead to large differences of results during acute study. Moreover, PW Doppler of LVOT flow and MA flow are obtained in a separate echocardiographic windows, the timing of the measurements are different and can introduce error. Current three-dimensional color Doppler-based methods require the operator to trace the area of color flow and use electrocardiographically gated three-dimensional echo over an acquisition of more than seven complete cardiac cycles. Furthermore, as arrhythmias and respiratory movement generate a stitching artifact, it is necessary to optimize acquisition via sinus rhythm gating and breath holding. At the same time, a novel real-time three-dimensional FVCD, based on instantaneous acquisition of a single cardiac cycle, has been reported as an innovative method to assess FMR [1] without significant manual interaction with the data post-processing. FMR quantification with three-dimensional FVCD showed better correlation and agreement than conventional two-dimensional methods. FMR was underestimated by two-dimensional methods, especially in multijet and dilated left ventricle. Multijet mitral regurgitation demonstrated a higher risk of discrepancy for the identification of surgical candidate, regardless of mitral regurgitation etiology [2]. This novel three-dimensional color Doppler flow quantification method based on instantaneous acquisition of a single cardiac cycle using a 4Z1c Matrix Array Transducer (Siemens Medical Solutions), which has 1728 elements, was used with the SC2000 system to obtain real-time non-gated three-dimensional volume and volume color Doppler images for this study. The three-dimensional data acquired included flow velocity information via color Doppler data, which have been made available by improvements in transducer and post-processing software technologies, to calculate mitral average regurgitation volume and stroke volumes semi-automatically from three-dimensional color Doppler data acquired at the mitral valve (MV) annulus and LVOT, with a wide-angle pyramidal volume. Previous two-dimensional and three-dimensional methods were not able to calculate stroke volumes at the MV and LVOT simultaneously. The data acquisition time was approximately 5 seconds for each three-dimensional color Doppler data set, and it took less than 3 minutes to analyze each stroke volume. The hemispheric program for sampling planes needed minimal manual adjustment by the operator for accurate analysis of cardiac output. It was demonstrated that measurement of flow volume through the MV by three-dimensional color Doppler echocardiography was correlated and agreed well with cardiac magnetic resonance imaging [1–3]. Gruner *et al.* have shown that the quantification of FMR before and after percutaneous MV repair by three-dimensional FVCD was comparable to the integrative visual assessment and more reliable than the PW Doppler method [4]. Kato *et al*. have described the accuracy and feasibility of computing mitral, aortic, tricuspid, and pulmonic stroke volumes using three-dimensional FVCD in children, to compute valve areas and regurgitant volumes [5]. These authors have concluded that this technique potentially provides a non-invasive alternative to historically invasively acquired hemodynamic data and to cardiac magnetic resonance imaging [5]. Intracardiac flow is a key physiological event that, in most instances, mediates the clinical consequences of anatomical perturbations. Three-dimensional echo is uniquely placed to overcome some of the well-known limitations of two-dimensional echo for flow quantification [6]. This is the first case report where automatic quantification of real-time three-dimensional FVCD has been proposed as a new, rapid, and accurate method for the assessment of FMR severity pre-CRT and post-CRT in combination with fluid dynamic echo-PIV approach.

Echo-PIV is an optical method where the contrast agent bubbles are tracked from one frame to the next to calculate the instantaneous blood velocity field. Echo-PIV has shown that regional anomalies of synchrony of the LV are related to the alteration of the physiological intracavitary pressure gradients that deviate from their natural longitudinal orientation [7, 8]. This deviation can be assessed by quantitative analysis of the orientation angle (*φ*) of the global hemodynamic forces exchanged between blood and surrounding tissues. Many echocardiographic studies have demonstrated how CRT can contrast all the pathophysiologic determinants of FMR by minimizing LV dyssynchrony due to the following: increasing “closing forces” (global synchronization), reducing “tethering forces” (local synchronization), reshaping annular geometry and function (local synchronization), and correcting diastolic mitral regurgitation (atrioventricular synchronization). The role for routine VV delay optimization post-CRT is not clear. Most studies have shown that a majority of patients have optimal VV intervals that are within a range of ±20 milliseconds. Simultaneous biventricular pacing or pre-excitation of LV most often remains a challenge. VV delay optimization is generally performed by changing the VV sequence, starting with the LV being activated before the RV, and then stepwise lengthening or shortening of the VV interval (for example, with intervals of 20 milliseconds) and measuring the highest aortic time-velocity integral (Ao IVT), but this simplified echocardiographic approach is susceptible to inter-observer variability because there is a significant manual interaction with the data post-processing. Furthermore, the availability of pacing configurations offered by quadripolar left ventricle leads could improve a patient’s response; however, selection of an optimal setting remains a challenge. Recent studies suggested that images of LV flow by echo-PIV could be a useful marker of synchrony [9]. The vortical hydrodynamic forces and their cytomechanical consequences by mechanosensing and mechanotransduction can radically affect ventricular remodeling with epigenetic nexus [10–12]. LV flow represents an integral outcome of the tissue contraction/relaxation process whose dynamic features (local and short lasting) may not be easily detectable in terms of tissue displacement. The fluid dynamics represents a sort of coupling between systole and diastole without a sharp separation between them [13], and the analysis of flow dynamics inside the LV can provide new information about LV systolic and diastolic function through the analytical representation of the distribution of intraventricular pressure gradients; this is because flow properties at one instant depend on the combination of mechanical events during previous time. The assessment of morphological and energetic characteristics of fluid dynamics, both at baseline pre-implantation and after biventricular pacing, is potentially combinable with three-dimensional FVCD to correct suboptimal device settings. We previously demonstrated that changes in electrical activation alter the orientation of blood flow momentum. The echo-PIV technique may be useful for elucidating the favorable effects of CRT on intraventricular fluid dynamics and it could be used to identify appropriate pacing setting during acute echocardiographic optimization of left pacing vector, with no relevant changes in electrical activation on electrocardiogram, and in PW Doppler mitral inflow or Ao IVT patterns. The long-term CRT outcome correlates with the degree of realignment of hemodynamic forces [7, 8, 14]. The CRT team consisting of experienced electrophysiologists and echocardiologists leads to improved patient outcomes, but current evidence does not strongly support the performance of atrioventricular and ventriculo-ventricular optimization routinely in all patients receiving CRT [15]. The applicability of dyssynchrony optimization in a “real-world” clinical setting is debated [16, 17], although sustained and effective biventricular pacing is crucial to achieving the best outcome from CRT. The degree of realignment of hemodynamic forces, with quantitative analysis of the orientation of blood flow momentum (*φ*), can represent improvement of fluid dynamics synchrony of the LV, and explain with a new deterministic parameter the effects of CRT on all the pathophysiologic determinants of FMR through the increase of “closing forces,” reduction of the “tethering forces,” a reshape of the annular geometry and function, and the correction of diastolic mitral regurgitation, respectively.

## Conclusions

This clinical case offers an innovative and accurate approach for acute echocardiographic optimization of left pacing vector. It shows the clinical utility of combined three-dimensional FVCD/echo-PIV assessment to increase response to CRT, in terms of reduction of FMR, improving fluid dynamics synchrony of the LV.
